# Ankyrin repeat and SOCS box containing protein 4 (Asb-4) colocalizes with insulin receptor substrate 4 (IRS4) in the hypothalamic neurons and mediates IRS4 degradation

**DOI:** 10.1186/1471-2202-12-95

**Published:** 2011-09-28

**Authors:** Ji-Yao Li, Biaoxin Chai, Weizhen Zhang, Xiaobin Wu, Chao Zhang, Danielle Fritze, Zefeng Xia, Cam Patterson, Michael W Mulholland

**Affiliations:** 1 Department of Surgery, University of Michigan, Ann Arbor, Michigan 48109 USA; 2 Carolina Cardiovascular Biology Center, University of North Carolina at Chapel Hill. North Carolina 27599, USA

## Abstract

**Background:**

The arcuate nucleus of the hypothalamus regulates food intake. Ankyrin repeat and SOCS box containing protein 4 (Asb-4) is expressed in neuropeptide Y and proopiomelanocortin (POMC) neurons in the arcuate nucleus, target neurons in the regulation of food intake and metabolism by insulin and leptin. However, the target protein(s) of Asb-4 in these neurons remains unknown. Insulin receptor substrate 4 (IRS4) is an adaptor molecule involved in the signal transduction by both insulin and leptin. In the present study we examined the colocalization and interaction of Asb-4 with IRS4 and the involvement of Asb-4 in insulin signaling.

**Results:**

*In situ *hybridization showed that the expression pattern of Asb-4 was consistent with that of IRS4 in the rat brain. Double *in situ *hybridization showed that IRS4 colocalized with Asb-4, and both Asb-4 and IRS4 mRNA were expressed in proopiomelanocortin (POMC) and neuropeptide Y (NPY) neurons within the arcuate nucleus of the hypothalamus. In HEK293 cells co-transfected with Myc-tagged Asb-4 and Flag-tagged IRS4, Asb-4 co-immunoprecipitated with IRS4; In these cells endogenous IRS4 also co-immunoprecipitated with transfected Myc-Asb-4; Furthermore, Asb-4 co-immunoprecipitated with IRS4 in rat hypothalamic extracts. In HEK293 cells over expression of Asb-4 decreased IRS4 protein levels and deletion of the SOCS box abolished this effect. Asb-4 increased the ubiquitination of IRS4; Deletion of SOCS box abolished this effect. Expression of Asb-4 decreased both basal and insulin-stimulated phosphorylation of AKT at Thr308.

**Conclusions:**

These data demonstrated that Asb-4 co-localizes and interacts with IRS4 in hypothalamic neurons. The interaction of Asb-4 with IRS4 in cell lines mediates the degradation of IRS4 and decreases insulin signaling.

## Background

The arcuate nucleus (ARC) of the hypothalamus is a key integrative center for peripheral and central signals controlling appetite and metabolism [1]. The ARC is ideally situated to sense peripherally circulating humoral factors as it is located in a site in the central nervous system with an impaired blood-brain barrier, and ARC neurons express high levels of CNS receptors for circulating factors including leptin and insulin [2,3] The best described anabolic neurons within the arcuate nucleus use neuropeptide Y and agouti-related peptide (NPY/AgRP) as neurotransmitters. Both NPY and AGRP stimulate food intake. A parallel and opposing pathway is comprised of neurons expressing proopiomelanocortin (POMC) and cocaine- and amphetamine-regulated transcript (POMC/CART). Both POMC and CART inhibit food intake. Efferent projections from NPY/AgRP and POMC/CART neurons to secondary energy homeostatic neurons in intra- and extra-hypothalamic sites are key features of energy homeostatic circuitry.

Asb-4 belongs to the ankyrin repeat and suppressor of cytokine signaling (SOCS) box containing protein family that has 18 members (Asb-1 to Asb-18). All Asb proteins have two functional domains, a C-terminal SOCS box region of approximately 40 amino acids and an up stream ankyrin repeat region [4]. The ankyrin repeat domain directs specific protein-protein interactions while the SOCS box region serves as an adapter to mediate the degradation of proteins targeted by the ankyrin repeat region [5-7]. The 18 Asb proteins vary in the numbers of ankyrin repeats, and contain other novel regions, corresponding to differing target proteins to which they bind. Asb-4 has nine ankyrin repeats N-terminal to its SOCS box [8]. Microarray analysis of ARC RNAs from fed and fasted rats revealed that Asb-4 was down-regulated by fasting. Quantitative PCR showed that Asb-4 mRNA was also down-regulated in the ARC of the genetically obese Zucker rat, a model of long-term energy imbalance. Expression of Asb-4 mRNA was restricted to neuroanatomical areas in the hypothalamus and amygdala associated with energy homeostasis. Notably, Asb-4 mRNA was expressed in the two types of ARC neurons critical to feeding behavior, POMC and NPY neurons [9]. However, the target(s) of Asb-4 in these neurons remains unknown.

Insulin receptor substrate (IRS) proteins represent a family of adapter proteins that play a central role in signal transduction by insulin, IGF-I, and a growing number of cytokines including leptin [10-12]. To date, six IRS proteins have been identified (IRS1-6) [13,14]. Although the different IRS proteins vary in size, they share a number of structural and functional characteristics. The NH_2 _-terminal of each protein consists of a homologous pleckstrin homology (PH) domain and a phosphotyrosine binding region (PTB). The PH and PTB domains mediate specific interactions with the insulin, IGF-I and cytokine receptors. The COOH-terminal portion of each protein contains a number of tyrosine phosphorylation sites [15,16]. Tyrosine phosphorylation sites in the C-terminal portion of IRS are phosphorylated upon the activation of receptors by hormones or cytokines. Tyr-phosphorylated IRS proteins serve as signaling scaffolds that propagate hormone action through binding of Src homology 2 (SH2) domain-containing proteins, such as the p85 regulatory subunit of PI3K [12].

The different members of IRS proteins have different tissue distributions. IRS4 mRNA is found to be highly expressed in the hypothalamus and amygdala of the brain [17]. However, the neuronal types that express IRS4 remain to be identified. In the present study we first examined whether POMC and NPY neurons express IRS4 mRNA. We then examined the colocalization and interaction of Asb-4 with IRS4 in neurons and the involvement of Asb-4 in the modulation of insulin signaling.

## Results

### Colocalization of Asb-4 with IRS4

**Figure 1:**
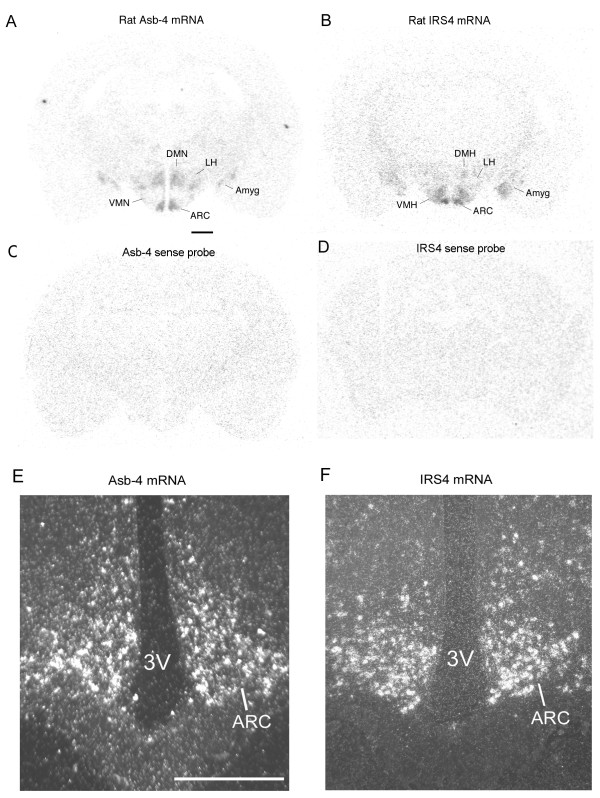
**Distribution of Asb-4 and IRS4 mRNAs in the rat brain**. Photographs are representatives of 15 brain sections from 5 animals for each hybridization. [^35^S]- labeled antisense or sense RNA probes were used for *in situ *hybridization. A. Antisense Asb-4 RNA probe. Scale bar = 1.0 mm. B. Antisense IRS4 RNA probe. C. Sense Asb-4 RNA probe. D. Sense IRS4 RNA probe. E. Dark field photomicrograph of Asb-4 mRNA in ARC. Scale bar = 0.5 mm. F. Dark field photomicrograph of IRS4 mRNA in ARC. ARC, arcuate nucleus; DMN, dorsomedial nucleus; LH, lateral hypothalamic area; VMH, ventromedial nucleus; Amyg, amygdala. 3V, third brain ventricle.

Using *in situ *hybridization IRS4 mRNA was found to be expressed in the arcuate nucleus, ventromedial nucleus (VMH), dorsomedial nucleus, lateral hypothalamus and amygdala, which is consistent with that reported by Numan et al [17]. Except for the VMH, which exhibits no Asb-4 expression, the expression pattern of Asb-4 was consistent with that of IRS4 (Figure 1A, B). Dark field photomicrograph showed that in the arcuate nucleus the expression pattern of Asb-4 was similar to that of IRS4 (Figure 1E, F).

**Figure 2:**
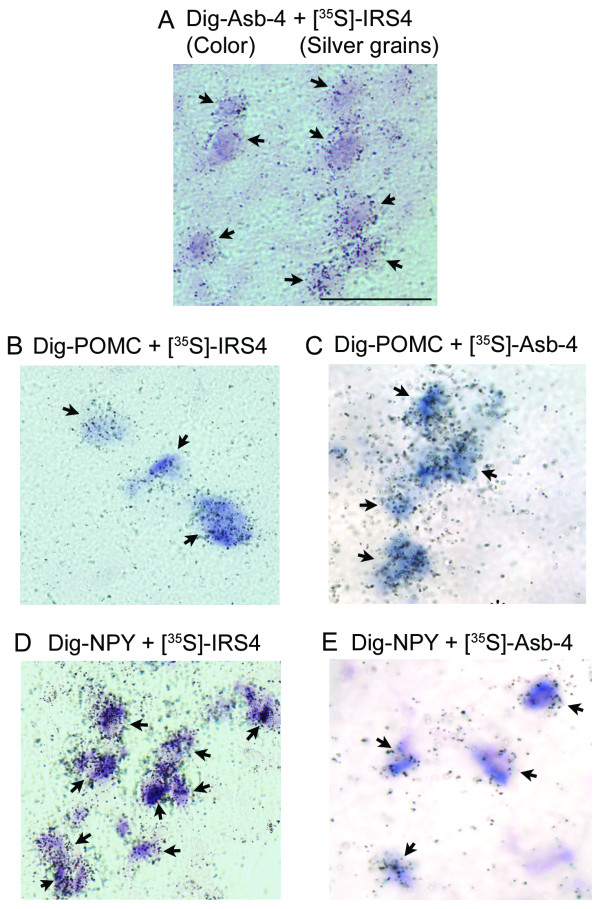
**Double *in situ *hybridization**. Photomicrographs are representatives of 15 brain sections from 5 animals for each colocalization. A. Purple color represents Asb-4 mRNA. Silver grains indicate IRS4 mRNA. B. Purple color represents POMC mRNA. Silver grains indicate IRS4 mRNA. C. Purple color represents POMC mRNA. Silver grains indicate Asb-4 mRNA. D. Purple color represents NPY mRNA. Silver grains indicate IRS4 mRNA. E. Purple color represents NPY mRNA. Silver grains indicate Asb-4 mRNA. Scale bar = 50 μm.

Figure 2 is a photomicrograph of double *in situ *hybridization in the arcuate nucleus. Fifteen coronal brain sections that cover the anterior, middle and posterior portions of the hypothalamus from 5 rats were hybridized and counted for each colocalization. Except for neurons within the VMH, almost all (98 ± 2%) Asb-4 positive cells (Figure 2A, purple color) have clusters of silver grains (IRS4) over them in the arcuate nucleus, dorsomedial nucleus, lateral hypothalamus and amygdala, indicating that Asb-4 colocalizes with IRS4. We next focused on the POMC and NPY neurons in the arcuate nucleus and determined if these neurons express IRS4 and Asb-4. As shown in Figure 2B and 2D, both POMC and NPY neurons (purple color) have dense clusters of silver grains (IRS4) over them, indicating that IRS4 was expressed in these neurons. Overall, 91 ± 4% of POMC and 93 ± 3% of NPY neurons express IRS4. In addition, 95 ± 1% of POMC neurons and 52 ± 2% of NPY neurons also express Asb-4 mRNA (Figure 2C and 2D).

### Interaction of Asb-4 with IRS4

**Figure 3:**
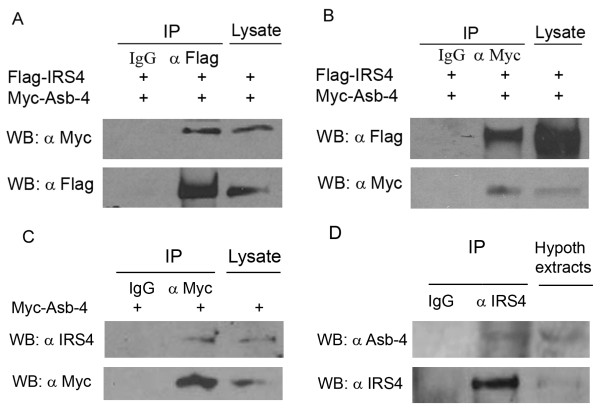
**Co-immunoprecipitation of IRS4 with Asb-4 in HEK293 cells and rat hypothalamus**. Flag-IRS4 and Myc-Asb-4 vectors were co-transfected into HEK293 cells. Cell lysates were precipitated with anti-Flag (A) or anti Myc (B) antibody-conjugated agarose, and blotted with either anti-Myc or anti-Flag antibody as indicated. C. Myc-Asb-4 vectors were transfected into HEK293 cells. Cell lysates were precipitated with anti Myc antibody-conjugated agarose and blotted with either anti IRS4 or anti Myc antibody. D. Rat hypothalamic extracts was precipitated with anti IRS4 antibody and blotted with either anti Asb-4 or anti IRS4 antibody. Normal IgGs and protein A beads were used as negative control in every experiment. All co-immunoprecipitation experiments were repeated three times.

Colocalization of Asb-4 with IRS4 in the hypothalamus implies that these molecules may have functional interactions. In order to examine this possibility we first performed co-immunoprecipitation (Co-IP) experiments using HEK293 cells. Myc-tagged full length mouse Asb-4 (Myc-Asb-4) and Flag-tagged full length mouse IRS4 (Flag-IRS4) were co-expressed in HEK293 cells and co-immunoprecipitated using either anti-Flag or anti-Myc antibody conjugated to agarose. As shown in Figure 3A, Myc-Asb-4 co-precipitated with Flag-IRS4 when Flag-IRS4 was precipitated with anti-Flag antibody, and Myc-Asb-4 was detected with anti-Myc antibody by Western blotting; Similarly Flag-IRS4 co-precipitated with Myc-Asb-4 when Myc-Asb-4 was precipitated with anti-Myc antibody, and Flag-IRS4 was detected with anti-Flag antibody (Figure 3B). Normal IgG was used as a negative control. These results demonstrate that Asb-4 interacts with IRS4 in HEK293 cells.

It has been reported that HEK293 cells have endogenous IRS4 expression [18]. We further examined the interaction between Asb-4 and IRS4 by co-immunoprecipitating endogenous IRS4 with transfected Myc-Asb-4 in these cells. When Myc-Asb-4 was precipitated with Myc antibody, IRS4 could be detected with anti IRS4 antibody (Figure 3C). These results showed that Myc-Asb-4 interacted with endogenous IRS4 in HEK cells.

Finally we performed co-immunoprecipitation with rat hypothalamic extracts to confirm the interaction *in vivo *since the transfected experiments have limitations. As shown in Figure 3D, when IRS4 was precipitated with anti IRS4 antibody, Asb-4 could be detected using anti Asb-4 antibody, indicating that Asb-4 interacts with IRS4 in the rat hypothalamus.

### Effect of Asb-4 on the levels, ubiquitination of IRS4 and insulin signaling

**Figure 4:**
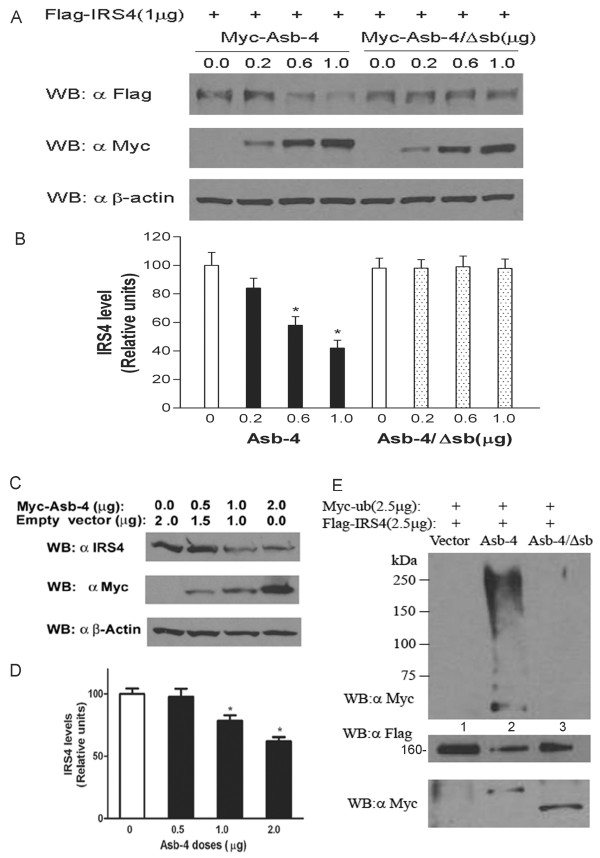
**Effect of Asb-4 on protein levels and ubiquitination of IRS4**. A.HEK293 cells were transfected with 1 μg (35 mm dish) Flag-IRS4 vector and differing amounts of Myc-Asb-4 or Myc-Asb-4/Δsb vector. The total amount of DNA in each transfection was equalized with pCMV empty vector. Cell lysates were first blotted with anti Flag antibody. The membranes were then stripped and reblotted with anti-Myc and anti-β-actin antibodies, respectively. B. Densitometry analysis of three separate experiments (n = 3). *p < 0.05 vs. control. C. HEK cells were transfected with Myc-Asb-4 vector and the total amount of DNA in each transfection was equalized with empty vector. D. Densitometry analysis of three separate experiments (n = 3). *p < 0.05 vs. control. E. Ubiquitination of IRS4 by Asb-4. CHO cells were co-transfected with Myc-ubiquitin, Flag-IRS4 and empty vector (Lane 1), or Myc-Asb-4 (Lane 2), or Myc-Asb-4/Δsb (Lane 3). Cell lysates were immunoprecipitated with anti-Flag conjugated agarose and first blotted with anti-Myc (top panel), and then the membrane is stripped and blotted with and Flag antibody (middle panel). The cell lysates were blotted with anti Myc antibody to show the expression of Myc-Asb-4 and Myc-Asb-4/Δsb (bottom panel).

We next examined the effect of Asb-4 expression on IRS4 levels. When Myc-Asb-4 and Flag-IRS4 were co-expressed in HEK293 cells, Asb-4 expression reduced IRS4 protein levels dose-dependently. Deletion of the SOCS box region (Asb4/Δsb) abolished the ability of Asb-4 to affect IRS4 levels (Figure 4A and 4B). When Asb-4 was over expressed in these cells it also decreased endogenous IRS4 levels dose-dependently (Figure 4C and 4D).

The general model of action of Asb proteins involves ubiquitination of proteins targeted by the ankyrin repeat region of the molecule. We next examined the effects of Asb-4 on ubiquitination of IRS4. As shown in Figure 4E, using Chinese hamster overy (CHO) cells, Asb-4 increased the ubiquitination of IRS4. Deletion of the SOCS box region abolished this effect.

**Figure 5:**
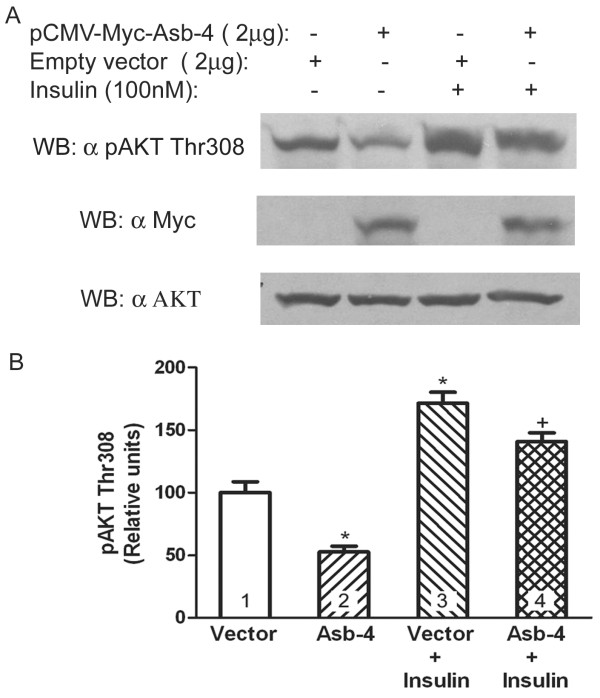
**Effect of Asb-4 on basal and insulin stimulated phospho AKT**. HEK cells were transfected with Myc-Asb-4 or empty vector. Cell lysates were blotted with antibodies as indicated. The membranes were stripped and reblotted with anti-Myc and anti-AKT antibodies, respectively (A). B. Densitometry analysis of three separate experiments (n = 3). *p < 0.05 vs. control (group 1); +p < 0.5 vs. group 3.

Since IRS4 has been reported to be involved in the insulin signaling pathway [19], and our data showed that Asb-4 decreased IRS4 level, we next examined the effect of Asb-4 on down-stream signaling of ISR4. In HEK293 cells expression of Asb-4 decreased both basal and insulin-stimulated phosphorylation of AKT at Thr308 (Figure 5).

## Discussion

The results of the current study demonstrate that insulin receptor substrate 4 co-localizes with Asb-4 within areas of the hypothalamus and amygdala that regulate caloric intake and energy expenditure. Asb-4 regulates IRS4 levels by directing its degradation via ubiquitination and decreasing the down-stream signal of IRS4. Five separate observations support this conclusion: 1) Asb-4 and IRS4 are both expressed in the arcuate nucleus, the dorsomedial nucleus of the hypothalamus and the amygdala; 2) Asb-4 colocalizes with IRS4, and both molecules are expressed in POMC and NPY neurons of the hypothalamus; 3) the molecules co-precipitate *in vitro *and *in vivo*; 4) increased expression of full-length Asb-4 causes decreased levels of IRS4; 5) expression of full-length Asb-4 increases ubiquitination of IRS4 and decreased phospho-AKT at Thr308.

Although IRS4 is thought to have a role in signal transduction similar to other member of the IRS family, the tissue distribution of IRS4 is substantially different than that of other IRS molecules. The restricted expression of IRS4 mRNA in the hypothalamus of the brain implies that IRS4 might be involved in the regulation of energy homeostasis. The current study further demonstrated that IRS4 is expressed in both NPY and POMC neurons of the arcuate nucleus. These two types of neurons regulate food intake and metabolism when activated by insulin and leptin [20,21]. The expression of IRS4 in NPY and POMC neurons implies that IRS4 may function to mediate the action of insulin and leptin in these neuronal types. The co-localization of Asb-4 and IRS4 provides an opportunity for these molecules to interact and modulate the signal transduction of insulin and/or leptin.

The co-expression of Asb-4 and IRS4 in amygdala is worth noting. The amygdala is primarily involved in emotions such as fear, anger and pleasure, memory etc [22]. The amygdala may also be involved in the role of motivation and reward in food intake [23]. The amygdala sends projections to the lateral hypothalamic area [24], which forms part of the feeding circuit and has been linked to initiation of feeding [25]. The neuronal type(s) that express Asb-4 and IRS4, and the roles these two proteins play in amygdala remain unknown.

Ankyrin repeat and SOCS box-containing protein-4 (Asb-4) was originally identified by a database search using a SOCS box consensus sequence [8]. There are five major protein families that have SOCS boxes. They differ in the type of domain that is upstream of the SOCS box. While several Asb proteins have been identified within the setting of a physiological process, no common functional theme is obvious. In the context of metabolic regulation, SOCS-3 down-regulates leptin receptor and insulin receptor signaling [26]. Brain-specific SOCS-3 knockout mice showed elevated leptin sensitivity, were resistant to high fat diet induced obesity and had attenuated insulin resistance [27]. SOCS-3 belongs to the SH2 domain protein family. Asb-6 is an adipocyte-specific protein that modulates APS interaction with the insulin receptor [6]. Utilizing a transgenic approach, we have recently reported that over-expression of Asb-4 in hypothalamic POMC neurons of mice produced a lean phenotype by increasing locomotive activity and metabolic rate in spite of the fact that the transgenic animals consumed significantly more food than wildtype littermates [28]. POMC-Asb-4 mice had higher rates of oxygen consumption and output of carbon dioxide than wildtype littermates, indicating that over expression of Asb-4 in POMC neurons increased energy expenditure. Consistent with the lean phenotype, blood leptin levels were lower in transgenic mice. The cellular basis for these observations is currently unknown and prompted a search for potential protein binding partners for Asb-4. In the current study, insulin receptor substrate-4 was observed to co-precipitate with Asb-4. Asb-4 increased degradation of IRS4 via ubiquitination in cell lines. Like all IRS family members, IRS4 acts as an adaptor and is involved in the signal transduction of insulin, leptin and other cytokines and growth factors. In HEK cells and neuronal N38 cells, exposure to insulin or leptin has been reported to increase tyrosine phosphorylation of IRS4 [11,18]. IRS4 interacts with the regulatory p85 subunit of phosphatidylinositol 3-kinase which then activates its downstream signal, AKT/PKB [19,29,30]. The inhibition of insulin signaling by Asb-4 via interacting with IRS4 may partially explain the hyperphagia in the POMC-Asb-4 transgenic mice since insulin can inhibit food intake in the brain.

All members of Asb protein family have two basic domains: the ankyrin repeat and the SOCS box. The ankyrin repeat interacts specifically with targeted proteins. The SOCS box region binds Elongin BC in a protein complex [31]. This protein complex has E3 ubiquitin ligase activity that facilitates polyubiquitination and proteasomal degradation of proteins bound by the upstream regions of the SOCS proteins [4]. Asb-4 is expected to follow this mechanism of action to increase ubiquitination of its target(s). The current study showed that Asb-4 interacts with IRS4, increases IRS4 ubiquitination and decreases IRS4 protein levels, probably by increasing its degradation. Deletion of the SOCS box of Asb-4 abolishes both the decrement in IRS4 levels and ubiquitination of IRS4, indicating that the SOCS box plays a key role in leading IRS4 to the degradation pathway. These observations are consistent with the general model of action of Asb proteins. The action of Asb-4 to direct degradation of IRS4 may be a mechanism by which NPY and POMC neurons modulate the sensitivity to circulating levels of insulin.

## Conclusions

This study demonstrated that Asb-4 co-localizes and interacts with IRS4 in hypothalamic neurons. The interaction of Asb-4 with IRS4 in cell lines mediates the degradation of IRS4 and decreases insulin signaling.

## Methods

### Antibodies and reagents

Anti-Myc antibody-conjugated agarose was purchased from Bethyl Laboratories (Montgomery, TX). Anti Myc monoclonal antibody was purchased from BD Bioscience (Palo Alto, CA). Anti-FLAG-M2 antibody, antibody-conjugated agarose and anti β-actin antibody were from Sigma-Aldrich (St. Louis, MO). Rabbit anti IRS4 polyclonal antibody was from Santa Cruz (Santa Cruz, CA) and mouse anti human IRS4 monoclonal antibody (M01) was from Abgent (San Diego, CA). Anti Asb-4 antibody was from Abcam Inc. (Cambridge MA). Anti phospho-AKT at Thr308 and total AKT was from Cell Signaling Technology (Beverly, MA).

### *In situ *hybridization

All studies were approved by the University of Michigan Committee on Use and Care of Animals.

A 568 bp fragment of the rat Asb-4 cDNA (codon 681 to 1248) was generated by PCR and subcloned into pBluescript SK (Stratagene, La Jolla, CA) at BamH I and EcoR I sites. The sense primer was: 5' CGG GAT CCG GAG CAG GAG TAC AGC AGG GAA CA 3'. The antisense primer was 5' CGG AAT TCT GAC AGT GGG AGG GAC AGC ATA GG 3'. A 566 bp fragment of the rat IRS4 cDNA (codon 3112 to 3677 according to gene bank number XM_001056753 was generated by PCR and subcloned into pBluescript SK at BamH I and Hind III sites. The sense primer was: 5' CCCCCAATGAACCAGGCTAAG 3'. The antisense primer was 5' ACAGGTGGTGGTTCGGAGTATCG 3'. The NPY cDNA (AI045437) in pT7T3D-PAC was purchased from Invitrogen (Carlsbad, CA). The rat POMC plasmid construct consisted of an 833 bp insert that included the full coding region of the POMC gene inserted into pGEM4Z (Promega, Madison, WI). The POMC-pGEM4Z plasmid was constructed by R. C. Thompson. The [^35^S]-labeled antisense and sense IRS4 and Asb-4 RNA probes, Dig-labeled antisense and sense Asb-4, NPY and POMC RNA probes were generated using standard *in vitro *transcription methodology [32].

Seven week old male Sprague-Dawley rats (280-300 g) were anesthetized with ketamine/xylazine and perfused via the ascending aorta with 200 ml of phosphate buffered saline (PBS), followed by 200 ml of 4% paraformaldehyde in PBS. The brain was postfixed for 16 h in 4% paraformaldehyde and then transferred to 20% sucrose (20% sucrose in PBS with 0.02% sodium azide) for 5 days at 4°C. The brain was embedded with 20% sucrose and Tissue-Tek O.C.T. (2:1) and coronal sections of 14 μ were cut on a cryostat. The sections were dried overnight at room temperature and were stored at -80°C until further processing. Sections studied covered the telencephalon and diencephalon from coordinates of bregma -0.80 mm to bregma -4.16 mm according to Paxinos and Watson [33].

*In situ *hybridization was conducted using a modification of a previously described technique [34,35]. The brain sections were washed 3 times with 2 × SSC and then digested with 0.45 μg/ml proteinase K (Invitrogen, Carlsbad, CA) in 100 mM Tris, pH 8.0, 50 mM EDTA) for 15 minutes at 37°C. After brief washing with distilled water, the sections were acetylated with 0.25% acetic anhydride in 0.1 M triethanolamine, pH 8.0, for 10 min. The sections were subsequently dehydrated through a graded series of ethanols.

Antisense or sense probes were diluted in hybridization buffer (50% formamide, 3 × SSC, 1 × Denhart's, 200 μg/ml yeast tRNA, 50 mM phosphate buffer, pH 7.4, 10% dextran sulfate and 10 mM DTT) to yield ≈ 30,000 cpm/μl. 40 μl diluted probes were applied to each slide and the sections were coverslipped. Slides were then placed in sealed plastic boxes lined with filter paper moistened with 50% formamide. The boxes were wrapped with plastic wrap and incubated at 55°C for 16 h. For dual label *in situ *hybridization, the sections were hybridized with antisense Dig-labeled Asb-4, NPY or POMC and [^35^S]-labeled IRS-4 riboprobes.

Following overnight incubation, coverslips were removed by dipping in 2 × SSC and the slides were washed with 2 × SSC. Slides were then incubated with 40 μg/ml RNase A (Roche; Indianapolis, IN) in 10 mM Tris-HCl (pH 8.0, 0.5 M NaCl) at 37°C for 1 h. The slides were washed with 2 x, 1 x, 0.5 × and 0.1 × SSC at room temperature for five minutes each time and then incubated in 0.1 × SSC at 67°C for 1 h. The sections were washed briefly with distilled water. For single label *in situ *hybridization the slides were dehydrated in graded alcohols and air-dried. Dried slides were exposed to Kodak BioMax film. For dual label *in situ *hybridization, after washing with distilled water, the sections were incubated with anti-Dig antibody (Roche, Indianapolis, IN) conjugated with alkaline phosphatase (1:20,000) overnight at room temperature. The slides were next incubated in nitroblue terazolium chloride and 5-bromo-4-chloro-3-indolyl-phosphate solution for 2-4 h for color reactions. Then the slides were dipped in ILFORD K.5D nuclear emulsion and exposed in dark for 14 days at 4°C. Sections were developed with D-19 developer.

### Co-immunoprecipitation

The full length coding region of mouse Asb-4 cDNA was cloned by RT-PCR from the mouse hypothalamic neuronal cell line, GT1-1, and subcloned into the pCMV-Myc vector (BD Clontech, Palo Alto, CA) using EcoR1 and Not I sites. The Myc tag was fused to the N-terminus of Asb-4. The correct frame of the construct was confirmed by DNA sequencing. Mouse IRS4 cDNA with full length coding region was amplified with the Expand Long Template PCR System (Roche, 68298 Mannheim, Germany) from C57BL genomic DNA since the full length coding region is encoded by a single exon. The cDNA was subcloned into 3xFlag CMV-10 vector (Sigma-Aldrich) at Hind III and Xba I sites. The 3xFlag was fused to the N-terminus of mouse IRS4. The correct frame of the construct was confirmed by DNA sequencing.

HEK293 cells were grown in Dulbecco's modified Eagles medium (DMEM) supplemented with 10% fetal bovine serum and 100 units/ml of penicillin and 100 μg/ml of streptomycin in a 37°C incubator supplemented with 5% CO_2_. Cells were transfected with 2.5 μg (100 mm dish) of pCMV-Myc-Asb-4 plasmid DNA along with 2.5 μg of p3xFlag-IRS4-CMV-10 plasmid DNA using lipofectamine 2000 as described by the manufacturer (Invitrogen). Forty hours after transfection, cells were disrupted by sonication in lysis buffer [150 mM NaCl, 20 mM Na_2_HPO_4_, pH 7.4, 1% Triton X-100 supplemented with protease inhibitor cocktail (Sigma)] and Flag-IRS4 or Myc-Asb-4 were immunoprecipitated using anti Flag-M2 or anti-Myc antibody conjugated agarose. After extensive washing the beads were resuspended in SDS-PAGE loading buffer and heated at 80°C for 5 min. The protein was resolved with SDS-PAGE gel and transferred to polyvinylidene membranes. The membranes were blotted with either anti-Myc, or anti Flag antibody.

For the co-immunoprecipitation of endogenous IRS4 from HEK cells, the cells were transfected with 5 μg Myc-Asb-4 vector (100 mm plate). Myc-Asb-4 was precipitated with anti-Myc antibody conjugated agarose and IRS4 was detected with anti human IRS4 monoclonal antibody (Abgent).

In order to confirm the interaction between Asb-4 and IRS4 *in vivo *twenty hypothalami were collected from adult rats (250-300 g body weight), homogenized in lysis buffer, centrifuged at 14,000 × g for 10 min at 4°C and the supernatant was used for immnoprecipitation with anti IRS4 antibody (Santa Cruz). Asb-4 was detected with anti Asb-4 antibody.

For the insulin stimulation of HEK cells, the cells were cultured in serum free DMEM with 0.1% BSA overnight; insulin was added to the cells and incubated for 20 min. The cells were harvested in lysis buffer and sonicated. Proteins were subjected to SDS-PAGE and transferred to polyvinylidene membranes.

### Ubiquitination assay

CHO cells co-transfected with pRK5 myc-ubiquitin, which is provided by Dr. Liangyou Rui (Department of Molecular and Integrative Physiology, University of Michigan), Flag-IRS4, and empty vector, or Myc-Asb-4, or Myc-Asb-4/Δsb. Forty hours after transfection, cells were disrupted by sonication in lysis buffer. The supernatants (cell lysates) were immunoprecipitated with anti-Flag conjugated agarose and blotted with anti-Myc antibody. Membranes were stripped and reblotted with anti-Flag antibody.

### Western blotting

Equal amounts of protein were resolved on SDS-PAGE gel, transferred to polyvinylidene membranes and immunoblotted with primary antibodies. Horseradish peroxidase conjugated secondary antibodies were used to detect antigen-antibody complexes via the ECL detection system (Amersham Biosciences, Piscataway, NJ).

### Statistical analysis

The density of bands was analyzed and normalized with internal controls (β-actin or total AKT) using the Kodak Gel Logic 440 Imaging System All. Values are expressed as mean ± SEM. Analysis of variation (ANOVA) followed by post Bonferroni test was used for statistical analysis. Significance was accepted as P < 0.05.

## List of abbreviations

ARC: arcuate nucleus; SOCS: suppressor of cytokine signaling; Asb-4: ankyrin repeat and SOCS box-containing protein 4; Asb-4/Δsb: Asb-4 minus its SOCS box; IRS4: insulin receptor substrate 4; POMC: pro-opiomelanocortin; NPY: neuropeptide Y; HEK293 cell: human embryonic kidney 293 cell; Co-IP: co-immunoprecipitation; SDS: sodium dodecyl sulfate; PAGE: polyacrylamide gel electrophoresis.

## Competing interests

The authors declare that they have no competing interests.

## Authors' contributions

JYL, CP and MWM conceived the study, participated in the experiment design and draft the manuscript; JYL and BX performed the experiments and data analysis; WZ, XW and ZX participated in the data analysis and manuscript revision. CZ and DF performed the experiments and data analysis. All authors read and approved the final manuscript.
